# Dysregulated serum chloride and clinical outcomes in critically ill adults: A systematic review and meta-analysis

**DOI:** 10.1371/journal.pone.0337560

**Published:** 2025-12-01

**Authors:** Xiaoliang Wan, Feiyao Deng, Xue Bai, Chenxi Xiang, Chuan Xu, Linxiao Qiu

**Affiliations:** 1 Department of Respiratory and Critical Care Medicine, West China Hospital, Sichuan University, Chengdu, Sichuan, China; 2 Department of Obstetrics, Sichuan Provincial Maternal and Child Health Hospital, Chengdu, Sichuan, China; 3 Operating Room, Chengdu Public Health Clinical Center, Chengdu, Sichuan, China; 4 The Third Department of Blood Collection, Chengdu Blood Center, Chengdu, Sichuan, China; 5 Department of Infectious Diseases, Luzhou City People’s Hospital, Luzhou, Sichuan, China; Rivers State University, NIGERIA

## Abstract

Dysregulated serum chloride levels are prevalent in critically ill patients. However, their clinical impact remains unclear. This first systematic review and meta-analysis quantified the prevalence of hypochloremia and hyperchloremia, and their associations with mortality and acute kidney injury (AKI) in critically ill populations. We searched PubMed, Embase, Web of Science, and the Cochrane Library for studies reporting hyperchloremia prevalence or outcomes in adult ICU patients until August 2025. Statistical analyses were conducted using Stata v16.0, and study quality was assessed using the Newcastle-Ottawa Scale. 34 studies (n = 175,021 patients) were included. The aggregated prevalence of hyperchloremia was 34% (95% CI [26%−43%]) and hypochloremia was 14% (95% CI [1%−28%]). Meta-analysis demonstrated that both hyperchloremia and hypochloremia were significantly associated with increased mortality, conferring a 28% (OR = 1.28, 95% CI [1.08–1.52]) and 55% (OR = 1.55%, 95% CI [1.33–1.81]) elevated risk for mortality, respectively. Crucially, a dose-response analysis revealed a non-linear relationship between serum chloride levels and mortality, confirming that the risk is independently elevated at both extremes. Furthermore, hyperchloremia was linked to an increased risk of AKI (OR = 1.40, 95% CI [1.07–1.85]). These findings establish dysregulated serum chloride as a common and clinically significant biomarker, underscoring the necessity of monitoring and managing both high and low chloride levels in critically ill patients. Future large-scale studies are warranted to validate these results and elucidate the mechanistic pathways linking chloride dysregulation to such adverse outcomes.

## Introduction

Serum chloride, the predominant extracellular anion in the human body, accounts for approximately one-third of the extracellular fluid tonicity [1]. It plays a crucial role in maintaining various physiological functions, including preserving the integrity of the gastrointestinal tract, supporting kidney function, regulating body fluid distribution, and facilitating muscle contraction [2]. The 0.9% sodium chloride solution (saline) is still a commonly used resuscitation fluid and medication diluent for critically ill patients [3]. However, recent research has revealed potential risks associated with dysregulated serum chloride. Anomalies in serum chloride levels are common in critically ill patients, and both hypochloremia and hyperchloremia have been linked to higher mortality in intensive care unit (ICU) patients [4]. Hyperchloremia is a medical condition marked by abnormally high chloride ion levels in the blood, typically defined as exceeding 106 mmol/L. Reported prevalence rates among critically ill populations vary substantially, ranging from approximately 17% to 40.8%, depending on the specific patient cohort and definition used [5,6]. A recent prospective observational study found that temporary hyperchloremia can even occur in 75% of critically ill patients within the first 24 hours of their ICU stay [7]. The high prevalence of hyperchloremia is a major concern because it has adverse effects on blood pressure, renal blood flow, and fluid balance in animal models [8] and healthy human subjects [9,10]. Regarding the relationship between hypochloremia and mortality, several studies have suggested that hypochloremia with chloride levels lower than 98 mmol/L is strongly related to a lower survival rate than normal chloride levels in critically ill patients [11–13], suggesting that hypochloremia is an independent prognostic marker of mortality.

Furthermore, evidence suggests that hyperchloremia is linked to increased mortality rates and a higher incidence of acute kidney injury (AKI) in critically ill adults [6,14–16]. AKI poses a major threat to hospitalized patients, with a notably high incidence among critically ill patients. Approximately half of all critically ill patients may develop AKI [17], which affects 30–60% of this patient population, and is closely associated with acute morbidity and mortality [18]. Emerging studies indicate that the repercussions of AKI extend well beyond the acute phase, and patients may progress to chronic kidney disease (CKD), face heightened cardiovascular risks, experience recurrent AKI episodes, and endure elevated long-term mortality rates [19]. In recent years, comparative research has highlighted the risk associated with 0.9% saline and AKI [15,16,20–22], as well as the link between high chloride loads from volume resuscitation and increased mortality [23–26]. However, recent randomized controlled trials (RCTs) comparing balanced crystalloids with normal saline in ICU fluid therapy have not demonstrated significant differences in AKI incidence or mortality [27–29], presenting conflicting evidence.

Despite inconsistent findings in previous studies, to date no systematic review has comprehensively synthesized the association between dysregulated serum chloride levels and critical clinical outcomes, particularly mortality and AKI. Therefore, the present meta-analysis aimed to clarify this association by synthesizing cohort studies to: (1) quantify the prevalence of dysregulated serum chloride levels (both hyperchloremia and hypochloremia); and (2) evaluate their relationship with mortality and AKI in critically ill patients. Critically ill patients were defined as those admitted to an ICU who met at least one of the following validated criteria: the Sequential Organ Failure Assessment (SOFA), Acute Physiology and Chronic Health Evaluation (APACHE) II scores, or a requirement for advanced organ support (e.g., mechanical ventilation or vasopressor therapy). A secondary objective was to characterize the dose-response relationship between serum chloride levels and mortality risk.

## Methods

### Design

A systematic review and meta-analysis focusing on dysregulated serum chloride levels (both hyperchloremia and hypochloremia) in critically ill adult patients was conducted, with the findings reported in compliance with the Preferred Reporting Items for Systematic Reviews and Meta-Analysis (PRISMA) guidelines (S1 File) [30]. Prior to conducting database searches, the protocol was prospectively registered in the International Prospective Register of Systematic Reviews (PROSPERO, registration No. CRD420251138216).

### Search strategy

A comprehensive search was conducted across several electronic databases, namely PubMed, Embase, Web of Science, and the Cochrane Library until August 2025. In addition to database searches, reference lists from the included studies and pertinent systematic reviews were manually examined to identify any additional eligible studies. Search strategies that combined Medical Subject Headings (MeSH) and free-text keywords were determined through consensus among the review team. Keyword searches were confined to title and abstract fields, employing terms such as “hyperchloremia”, “hypochloremia”, “Chlorides”, “ICU”, “intensive care unit”, “intensive care units”, and “critically ill”. Boolean operators (OR, AND) were uniformly applied across all databases to merge the search terms. The detailed search strategies for each database can be found in S2 File. We also manually screened the reference lists of the retrieved articles to identify additional relevant studies.

### Inclusion and exclusion criteria

Studies were selected for inclusion based on the following criteria: (1) Population: Adult patients (≥ 18 years) admitted to a critical care unit; (2) Exposure: dysregulated serum chloride levels (hyperchloremia or hypochloremia); (3) Comparators: Patients with normal serum chloride levels; (4) Outcomes: the prevalence of hyperchloremia and hypochloremia, and the association between hyperchloremia/hypochloremia and the risk of AKI and mortality; (5) Study Design: Observational studies, including cohort, case-control, and cross-sectional designs.

The following criteria were applied for study exclusion: Studies that were unpublished, such as preprints and grey literature were excluded; non-original research, including reviews, guidelines, letters, and case reports, was not considered; Studies were also excluded if the full text was inaccessible, or if they contained inadequate outcome data. In cases of overlapping patient cohorts, only the study with the largest sample size was included. In details, potential overlaps were identified by comparing author affiliations, study periods, and recruitment windows. When duplication was suspected, we prioritized studies based on the following criteria: firstly, the completeness and relevance of outcome data; secondly, the comprehensiveness of cohort description and clinical details; and finally, the largest sample size when other factors were equivalent. This approach prevented double-counting while maximizing data quality and relevance for each analysis.

### Literature screening and data extraction

Duplicate records were removed using EndNote X9 software. The titles and abstracts of the studies were independently screened by two reviewers (Feiyao Deng and Xue Bai), followed by a full-text assessment using predefined inclusion and exclusion criteria. Any differences of opinion between the two reviewers were settled through discussion, and for cases that remained unresolved, a third reviewer (Xiaoliang Wan) made the final decision.

Data extraction was independently performed by two reviewers (Feiyao Deng and Xue Bai) using a pre-validated standardized form, with results cross-checked and tabulated. The information gathered included the first author’s name, publication year, country of origin, study design, characteristics of the ICU patient population, study duration, sample size, patient age and gender, and outcome indicators. Any disagreement was resolved by consensus or discussion with a third author (Xiaoliang Wan).

### Quality evaluation

The Newcastle-Ottawa Scale (NOS) was used to evaluate the methodological quality of all the studies included in the analysis [31]. The NOS criteria assess studies in three domains: cohort selection (scored 0–4 stars), comparability (0–2 stars), and outcome assessment (0–3 stars), culminating in a maximum total score of 9 stars. Each study was independently evaluated by two authors (Feiyao Deng and Xue Bai). Studies that attained an NOS score of 6 or higher were classified as high-quality. In cases where there were discrepancies in the assessments between the two authors, these were brought to the attention of a third researcher (Xiaoliang Wan) for discussion and resolution, thereby ensuring consistency in the evaluation process.

### Statistical analysis

Statistical analyses were carried out utilizing Stata version 16.0. The pooled prevalence estimates of hyperchloremia and hypochloremia were determined through a random-effects model. To compare chloride levels between survivors and non-survivors, standardized mean differences (SMDs) with 95% confidence intervals (CIs) were employed. For studies reporting multiple effect measures for the association between hyperchloremia/hypochloremia and the risk of mortality and AKI, we prioritized adjusted over unadjusted odds ratios (ORs) to account for potential confounding variables. These ORs were log-transformed for the meta-analysis, and the generic inverse variance method was used to compute pooled estimates, ensuring a consistent statistical approach. The results were illustrated using forest plots. The degree of heterogeneity among the studies was measured using the I^2^ statistic. An I^2^ value of 50% or higher indicated substantial heterogeneity, which warranted the use of the random-effects model; conversely, an I^2^ value below 50% indicated low heterogeneity. Where sufficient data were available, potential sources of heterogeneity were investigated through subgroup analyses and meta-regression, and the variables used for this purpose included population type (e.g., COVID-19 patients, CVD patients, postoperative patients, or critically ill patients in general), patient age (older than 60 vs. 60 years or younger), and the diagnostic threshold for hyperchloremia (higher than 106 mmol/L vs. higher than 110 mmol/L). Publication bias was evaluated using funnel plots and Egger’s test [32]. A *p*-value of less than 0.05 (in a two-tailed test) was regarded as statistically significant.

To elucidate the detailed dose-response relationship between chloride levels and mortality, we conducted a dose-response meta-analysis [33–35]. The analysis was performed according to the standard methodology, which necessitates data on the total number of participants, number of cases, and odds ratios with variances for at least three quantitative exposure categories. For each study, the median chloride level of each category was used; if not reported, the midpoint of the category boundaries was substituted. The width of any open-ended category was assumed to match that of the closest adjacent category [36]. Risk estimates were converted to a consistent reference when necessary [37]. We modeled the association using a restricted cubic spline model (knots at the 10th, 50th, and 90th percentiles) and assessed non-linearity by testing the significance of the second spline coefficient. The model used a serum chloride concentration of 106 mmol/L as the reference, a clinically relevant value within the normal range. All statistical tests were two-sided, with a significance threshold of *p* < 0.05.

## Results

### Identification of studies

**Fig 1:**
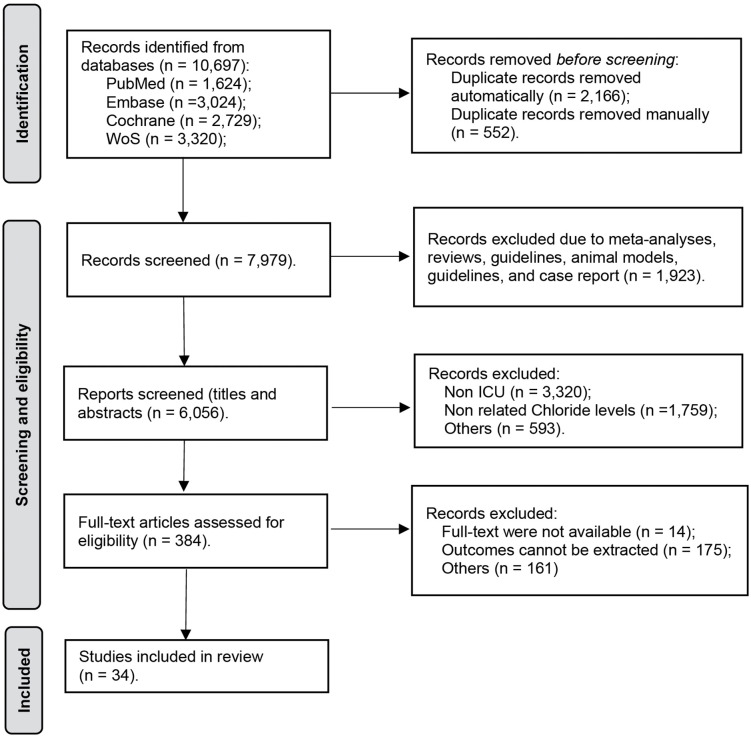
PRISMA flow diagram of the included and excluded articles.

The literature search process is presented as a PRISMA flow diagram in Fig 1. Initially, a total of 10,697 potentially relevant studies were automatically and manually identified from the four electronic databases mentioned above. 2,718 duplicate records were removed from the dataset, and the titles and abstracts of 7,979 records were screened. Among these, 1,923 records were excluded due to being ineligible publication types, such as previous meta-analyses, reviews, guidelines, animal studies, and case reports, or because they were clearly not relevant to the study. The remaining 6,038 records were then screened against the predefined inclusion and exclusion criteria, leading to the exclusion of 5,672 records. Full-text assessment was conducted on 366 articles, of which 34 met the inclusion criteria and were incorporated into the analysis.

### Study characteristics

The detailed characteristics of all studies included in the analysis are presented in Table 1. A total of 34 retrospective studies were examined [5,12,13,26,14,38−66], involving 175,021 patients. Among the analyzed studies, sample sizes varied from 62 [39] to 48,704 [59], and the mean age of patients ranged from 35.24 years [44] to 75.50 years [13]. Geographically, the patient populations in these studies were mainly from China [5,13,43,48,49,62–66], followed by the USA [41,45,14,52,54,55,59,60], and other Asian countries, with populations from Japan [12,57], Korea [26,46], India [39], and Saudi Arabia [51]. The remaining patient groups came from European countries [47,50,53,56,58,61] and African countries [38,40,42,44]. Regarding patient populations, 12 [42,56] studies [40,44,50,51,54,55,57,59,61,64] included critically ill patients without specifying their underlying cause, 6 studies focused on patients with cerebral pathologies, such as cerebral hemorrhage [41,46,52,63], cerebral injury [45,47], or hemispheric infarction [46], 4 studies enrolled patients with cardiovascular disease (CVD), including those with heart failure [13,62], and stroke [5,60], as well as 3 studies [12,26,58] included post-surgical critically ill patients, while the remaining studies centered on specific conditions like coronavirus disease 2019 (COVID-19) [38,39], sepsis [48,14,63], AKI [43,68], and cirrhosis [53], and Parkinson’s disease [49].

**Table 1:** Characteristics of the studies included in the systematic review.

Studies	Countries	Design	ICU patients	Data collect period	Samples size (n)	Gender F (%)	Age (mean)	Outcomes reported
Amara et al (2021)	India	Retrospective	COVID-19	2021	62	75.81	55.70	Rate^1^, in-hospital mortality
Barlow et al (2022)	USA	Retrospective	aSAH	2015-2019	234	41.00	55.00	Rate^1^
Gwak et al (2021)	Korea	Retrospective	LHI	2013-2018	90	50.00	71.00	Serum chloride levels, AKI
Huang et al (2018)	China	Retrospective	CVD (stroke)	2013-2016	405	69.75	59.13	Rate^1^, 30-day mortality
Kimura et al (2014)	Japan	Retrospective	Postoperative	2011-2015	98	65.50	74.50	Serum chloride levels
Lee et al (2016)	Austria	Retrospective	Major trauma	2011-2016	266	74.00	49.70	Rate^1^, Serum chloride levels
Oh et al (2018)	Korea	Retrospective	Postoperative	2011-2016	7,991	58.85	60.05	Rate^1^, AKI
Shao et al (2016)	USA	Retrospective	Critically ill	2006-2012	6,025	51.80	63.00	Rate^1^, AKI
Regenmortel et al (2016)	Belgium	Retrospective	Postoperative	2007-2017	10,165	62.00	62.70	Rate^1^, 30-day mortality, in-hospital mortality
Yeh et al (2020)	USA	Retrospective	Critically ill	2001-2012	48,704	56.30	65.70	Rate^1^, in-hospital mortality, AKI
Yessayan et al (2017)	USA	Retrospective	CVD (Septic shock)	2007-2012	6,490	51.47	66.57	Rate^1^, AKI
Zhang et al (2024)	China	Retrospective	CVD (Heart failure)	2008-2019	15,983	54.60	72.90	Rate^1^, in-hospital mortality
Zhu et al (2022)	China	Retrospective	AKI	2014-2015	4,234	57.01	65.78	In-hospital mortality, ICU mortality
Al Qahtani et al (2023)	Saudi Arabia	Retrospective	COVID-19	2020-2021	255	66.70	56.80	Rate^1^, mortality
Tan et al (2024)	China	Retrospective	CVD (Heart failure)	2008-2019	9,364	55.30	75.50	Rate^1^, mortality
Semmler et al (2023)	Austria	Retrospective	Cirrhosis	2003-2020	181	72.00	51.90	Rate^1^, ICU mortality
Ditch et al (2020)	USA	Retrospective	TBI	2009-2017	458	71.00	51.00	In-hospital mortality, serum chloride levels
Martin et al (2013)	Germany	Retrospective	Critically ill	2004-2007	1,551	53.97	58.60	Mortality
Yilman et al (2022)	Turkey	Retrospective	Critically ill	2017-2019	373	50.40	73.27	Rate^1^, mortality
Sadan et al (2017)	USA	Retrospective	SH	2009-2014	1,267	63.25	54.20	AKI
Badawy et al (2022)	Egypt	Retrospective	Critically ill	2019-2021	400	63.00	–	Mortality
Zhou et al (2023)	China	Retrospective	Critically ill	2020-2022	2,024	62.40	66.98	Rate, AKI
Vasconcellos et al (2018)	South Africa	Retrospective	Critically ill	2015-2016	250	56.00	35.24	AKI
Masevicius et al (2017)	Argentina	Retrospective	Critically ill	2006-2014	4,901	49.00	64.00	Serum chloride levels
Neyra et al (2015)	USA	Retrospective	Septic critically ill	2007-2012	1,940	51.25	66.45	Rate^1^, In-hospital mortality
Sen et al (2017)	USA	Retrospective	Critically ill	2000-2008	4,710	49.89	> 18	AKI, 30-day mortality,
Tani et al (2012)	Japan	Retrospective	Critically ill	2009-2012	488	60.00	61.80	Rate^1.2^, mortality, serum chloride levels
Zhou et al (2022)	China	Retrospective	IH	2001-2012	376	54.50	69.93	Rate^1^, mortality, serum chloride levels
Chen et al (2025)	China	Retrospective	AKI	2008-2022	19,107	64.36	43.06	Rate^1,2^, mortality
Lei et al (2025)	China	Retrospective	Septic critically ill	–	6,219	66.99	35.74	Rate^2^, mortality
Luo et al (2025)	China	Retrospective	PD	2008-2019	143	79.32	67.13	Mortality, serum chloride levels
Świstek et al (2025)	Poland	Retrospective	Critically ill	2020-2022	1,726	67.00	70.70	Serum chloride levels
Nechba et al (2025)	Morocco	Prospective	Critically ill	2019	798	57.30	51.90	Rate^1^, mortality
Zhao et al (2025)	China	Retrospective	Septic critically ill	2023	17,743	66.49	58.26	Rate^1^, mortality

Note. ICU, intensive care unit; aSAH, aneurysmal subarachnoid hemorrhage; LHI, large hemispheric infarction; AKI, acute kidney injury; TBI, traumatic brain injury; SH, Subarachnoid Hemorrhage; IH, intracerebral hemorrhage; AKI, acute kidney injury; PD, Parkinson’s disease; COVID-19, coronavirus disease 2019; CVD, cardiovascular disease. ^1^, the prevalence rate of hyperchloremia; ^2^, the prevalence rate of hypochloremia.

### Quality assessment and publication bias

According to the quality assessment using the NOS for the included studies, the results indicated that the overall quality of the studies was generally high. Regarding the overall scores, 3 studies (8.8%) achieved the highest score of 9 stars [14,45 ,61], 17 studies (50.0%) scored 8 stars [5,26,40–42,44,47,48,50,53–56,58,63,65,66], 11 studies (8.8%) scored 7 stars [13,38,39,43,46,49,51,52,57,61,62], and 3 studies scored 6 stars [12,59,64]. In details, 16 studies (47.1%) achieved the full score of 4 stars in the cohort selection domain, reflecting rigorous cohort design [14,26,40,41,44,45,47,48,53–55,58,60,63,65,66]. For comparability, the majority of studies (94.1%) scored 2 stars, indicating adequate control for confounding factors in most cases, while only two studies scored 1 star [51,59]. In the outcome assessment domain, scores were more varied, with 7 studies (20.6%) received 3 stars [5,14,42,45,51,56,60], 26 studies (76.5%) scored 2 stars [12,13,26,38–41,43,44,46–50,52–55,57–59,61–63,65,66], and 1 study (2.9%) scored 1 star [64]. Overall, the included studies demonstrated high quality, although certain studies still required optimization in the design of comparability or outcome assessment domains (Table 2).

**Table 2:** Risk of bias evaluation.

Studies	Cohort selection	Comparability	Outcome assessment	Total
Amara et al (2021)	★★★	★★	★★	7★
Barlow et al (2022)	★★★★	★★	★★	8★
Gwak et al (2021)	★★★	★★	★★	7★
Huang et al (2018)	★★★	★★	★★★	8★
Kimura et al (2014)	★★	★★	★★	6★
Lee et al (2016)	★★★★	★★	★★	8★
Oh et al (2018)	★★★★	★★	★★	8★
Shao et al (2016)	★★★★	★★	★★	8★
Regenmortel et al (2016)	★★★★	★★	★★	8★
Yeh et al (2020)	★★★	★	★★	6★
Yessayan et al (2017)	★★★★	★★	★★★	9★
Zhang et al (2024)	★★★	★★	★★	7★
Zhu et al (2022)	★★★★	★★	★★	8★
Al Qahtani et al (2023)	★★★	★★	★★	7★
Tan et al (2024)	★★★	★★	★★	7★
Semmler et al (2023)	★★★★	★★	★★	8★
Ditch et al (2020)	★★★★	★★	★★★	9★
Martin et al (2013)	★★★	★★	★★	8★
Yilman et al (2022)	★★★	★★	★★	7★
Sadan et al (2017)	★★★	★★	★★	7★
Badawy et al (2022)	★★★★	★★	★★	8★
Zhou et al (2023)	★★★	★★	★	6★
Vasconcellos et al (2018)	★★★★	★★	★★	8★
Masevicius et al (2017)	★★★	★	★★★	7★
Neyra et al (2015)	★★★★	★★	★★★	9★
Sen et al (2017)	★★★★	★★	★★	8★
Tani et al (2012)	★★★	★★	★★	7★
Zhou et al (2022)	★★★★	★★	★★	8★
Chen et al (2025)	★★★	★★	★★	7★
Lei et al (2025)	★★★★	★★	★★	8★
Luo et al (2025	★★★	★★	★★	7★
Świstek et al (2025)	★★★	★★	★★★	8★
Nechba et al (2025)	★★★	★★	★★★	8★
Zhao et al (2025)	★★★★	★★	★★	8★

### Meta-analysis results

#### The prevalence of hyperchloremia and hypochloremia in critically ill patients.

**Fig 2:**
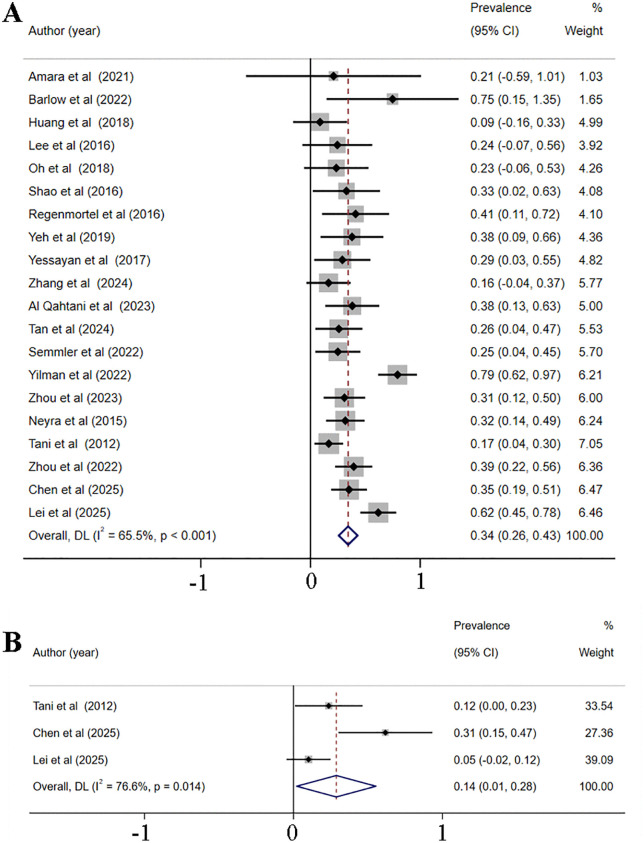
Meta-analysis results of overall prevalence of hyperchloremia (A) and hypochloremia (B).

20 studies [5,13,14,26,38,39,41,43,47,48,53,55,57–62,64,65], encompassing 124,035 critically ill patients, provided data on the prevalence of hyperchloremia, and 3 studies [43,48,57] with 25,290 critically ill patients assessed the prevalence of hypochloremia. The reported prevalence of hyperchloremia in these studies varied widely, ranging from 9% [5] to 79% [61]. A random-effects meta-analysis was conducted, which revealed a pooled prevalence of 34% (95% CI [26%−43%]; *p* < 0.001, I^2^ = 65.5%), as depicted in Fig 2A. Subgroup analyses were carried out based on the chloride threshold for diagnosing hyperchloremia (details can be seen in S3 File), patient age, and the type of ICU population to explore the prevalence of hyperchloremia. Notable differences in the prevalence of hyperchloremia were found among the subgroups. Specifically, the prevalence of hyperchloremia was 33% (95% CI [22%−44%], *p* < 0.001) with a chloride threshold over 106 mmol/L, while prevalence of hyperchloremia was only 27% (95% CI [19%−36%], *p* < 0.001) with the chloride threshold over 110 mmol/L. Meanwhile, patients under 60 years old had a hyperchloremia prevalence of 35% (95% CI [21%−50%], *p* < 0.001), and those over 60 years old had a prevalence of 34% (95% CI [23%−45%], *p* < 0.001). Among patients with CVD, the prevalence was 27% (95% CI [15%−39%], *p* < 0.001), whereas for critically ill patients in general, it was 38% (95% CI [18%−58%], *p* < 0.001). Significant differences in hyperchloremia prevalence were evident among subgroups categorized by a chloride threshold and ICU population type. The detailed subgroup results are presented in Table 3. Sensitivity analyses were performed but the source of heterogeneity was not identified (S1 Fig). The funnel plot (S2 Fig) and Egger’s test (*p* = 0.942) revealed no publication bias. Besides, the prevalence of hypochloremia was 14% (95% CI [1%−28%], *p* < 0.001, Fig 2B) among the included studies with no publication bias (*p* = 0.169).

**Table 3:** Subgroup analysis of the prevalence of hyperchloremia in critically ill patients.

Subgroups	Numbers of included studies	Heterogeneity		Meta-analysis related results	
		I^2^	*p*	Prevalence [95% CI]	*p*
**Chloride threshold**					
> 106 mmol/L	11	58.1%	0.008	33% [22%−44%]	< 0.001
> 110 mmol/L	7	0.0%	0.782	27% [19%−36%]	< 0.001
**Age**					
≤60	8	60.2%	0.014	35% [21%−50%]	< 0.001
> 60	12	70.1%	< 0.001	34% [23%− 45%]	< 0.001
**Population**					
COVID-19	2	0.0%	0.689	37% [13%−60%]	< 0.001
Critically ill	6	84.3%	< 0.001	38% [18%−58%]	< 0.001
CVD	4	35.1%	0.173	27% [15%−39%]	< 0.001
Postoperative	2	0.0%	0.415	32% [11%−53%]	< 0.001
Others	4	70.4%	0.017	38% [20%−57%]	< 0.001

COVID-19, coronavirus disease 2019; CVD, cardiovascular disease

### The chloride levels between survived and non-survived critically ill patients

**Fig 3:**
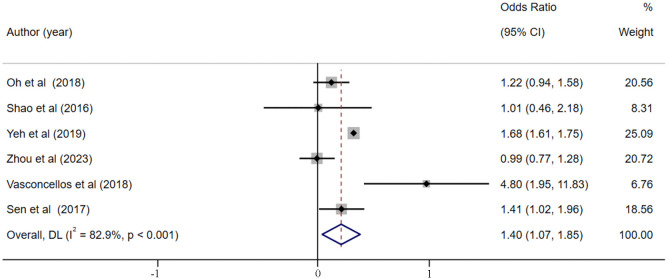
Forest plot of serum chloride levels between survived and non-survived critically ill patients.

8 studies [12,46,47,51,56,57,63,65], which included 17,891survivors and 7,797 non-survivors, presented data on the differences in averaged mean serum chloride levels between these two groups. Given the considerable heterogeneity observed among the studies (I^2^ = 97.0%, *p* < 0.001), a random-effects model was employed to pool the correlation effect values (Fig 3). The findings revealed that there was a significant difference in the averaged mean serum chloride levels between survivors and non-survivors (SMD = 0.40, 95% CI [0.13–0.67], *p *= 0.004). Sensitivity analyses did not identify any sources of heterogeneity (S3 Fig). Univariate meta-regressions to explore potential sources of heterogeneity depending on patient age but failed to detect the sources of heterogeneity (*p *= 0.497). According to Egger’s test and funnel plot, no publication bias was observed among the included studies (S4 Fig, *p* = 0.873).

### The impact of hyperchloremia and hypochloremia on the risk of mortality in critically ill patients

**Fig 4:**
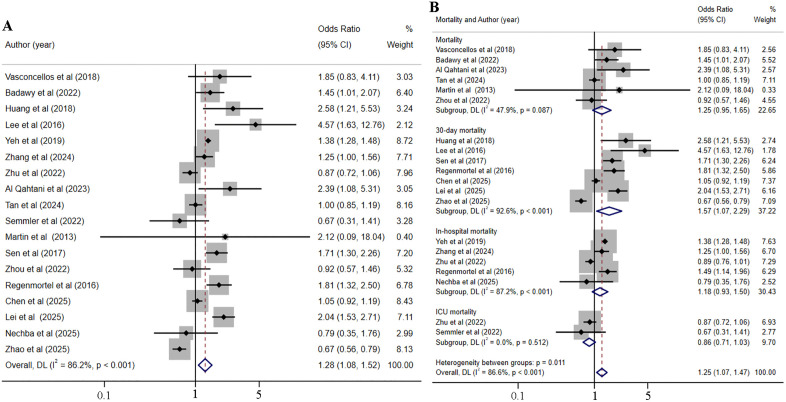
Forest plot of the association between hyperchloremia and the risk of mortality (A) and subgroup analysis of the association between hyperchloremia and the risk of mortality (B).

18 studies [5,13,38,40,42–44,47,48,50,54,55,60,63,64,66,67], involving 96,844 critically ill patients, were incorporated to evaluate the impact of hyperchloremia on mortality risk. Given the high heterogeneity among the included studies evaluating the impact of hyperchloremia on mortality risk (I^2 ^= 86.2%, *p* < 0.001), a random-effects model was adopted to pool the correlation effect values (Fig 4A). The analysis indicated that among critically ill patients, hyperchloremia was linked to a 28% increased risk of mortality (OR = 1.28, 95% CI [1.08–1.52], *p* = 0.005). Furthermore, subgroups were stratified based on the time or location of mortality, while the results demonstrated significant variations in the relationship between hyperchloremia and different mortality rates (Fig 4B). Specifically, hyperchloremia was associated with an increased risk of 30-day mortality (OR = 1.57, 95% CI [1.07–2.29], *p* = 0.021), but not with an increased risk of in-hospital mortality, ICU mortality, and overall mortality (without mention the specific time or location of mortality). The results showed that the time or location of mortality was the source of heterogeneity (ICU mortality, I^2^ = 0.0%, *p* = 0.512; In-hospital mortality, I^2^ = 87.2%, *p* < 0.001; 30-day mortality, I^2^ = 92.6%, *p* < 0.001). The sensitivity analysis showed that the results remained consistent after excluding any study, indicating stable and reliable results (S5 Fig). No publication bias was found among the included studies, as seen from the funnel plot (S6 Fig) and Egger’s test (*p* = 0.686).

**Fig 5:**
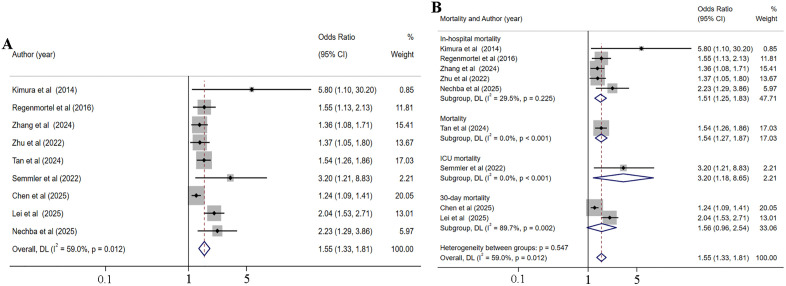
Forest plot of the association between hypochloremia and the risk of mortality (A) and subgroup analysis of the association between hypochloremia and the risk of mortality (B).

9 studies [12,13,42,43,48,53,58,62,66] were included to assess the impact of hypochloremia on mortality risk. Given the significant heterogeneity (I^2 ^= 59.0%, *p* = 0.012), a random-effects model was utilized to aggregate the effect estimates (Fig 5A). Among critically ill patients, the analysis indicated that hypochloremia was linked to a 55% increased risk of mortality (OR = 1.55, 95% CI [1.33–1.81], *p* < 0.001). Furthermore, subgroups were stratified based on the time or location of mortality, and the results (Fig 5B) showed that hypochloremia was only associated with an increased risk of in-hospital mortality (OR = 1.51, 95% CI [1.25–1.83], *p* = 0.021), but not with 30-day mortality. High heterogeneity was mostly observed the included studies assessing the risk of 30-day mortality (I^2^ = 89.7%, *p* = 0.001) not in in-hospital mortality (I^2^ = 29.5%, *p* = 0.225), suggesting the time or location of mortality might be the source of heterogeneity. The sensitivity analysis showed that the results remained consistent after excluding any study, indicating stable and reliable results (S7 Fig). According to the funnel plots (S8 Fig) and p values of Egger’s test, publication bias was observed (*p* = 0.005). Publication bias in this meta-analysis was evaluated using the trim-and-fill method with funnel plot visualization. After incorporating data from 3 theoretically imputed studies (as shown in S9 Fig), the pooled effect size was calculated as OR = 1.46 with 95% CI [1.24–1.73]. This outcome demonstrates that while publication bias was detected in the original studies, the trim-and-fill adjustment resulted in minimal changes to the effect estimates, indicating the relative robustness of the findings despite the presence of publication bias.

### The impact of hyperchloremia on the risk of AKI in critically ill patients

**Fig 6:**
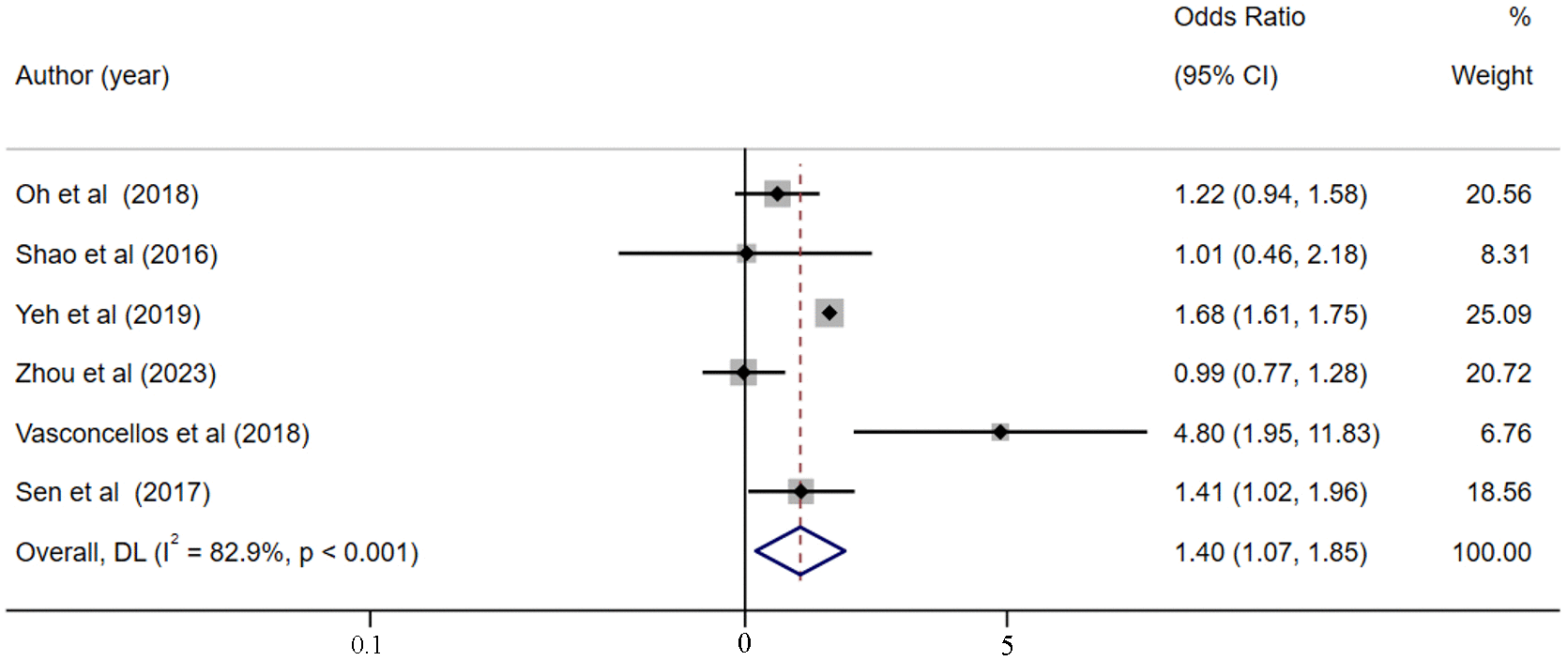
Forest plot of the association between hyperchloremia and the risk of AKI.

6 studies [26,44,54,55,59,64] explored the relationship between hyperchloremia and the risk of AKI in critically ill patients. Despite the presence of high heterogeneity (I^2 ^= 82.9%, *p* < 0.001, Fig 6), the pooled analysis with random-effects mode demonstrated that hyperchloremia was significantly associated with a 40% increase in AKI risk (OR = 1.40, 95% CI [1.07–1.85], *p *= 0.015). Sensitivity analysis did not identify the source of heterogeneity (S10 Fig), and no publication bias was observed according to the Egger’s test (*p* = 0.346). Univariate meta-regressions to explore potential sources of heterogeneity depending on patient age but failed to detect the sources of heterogeneity (*p *= 0.111).

### Dose–response analysis of serum chloride levels and mortality risk in critically ill patients

**Fig 7:**
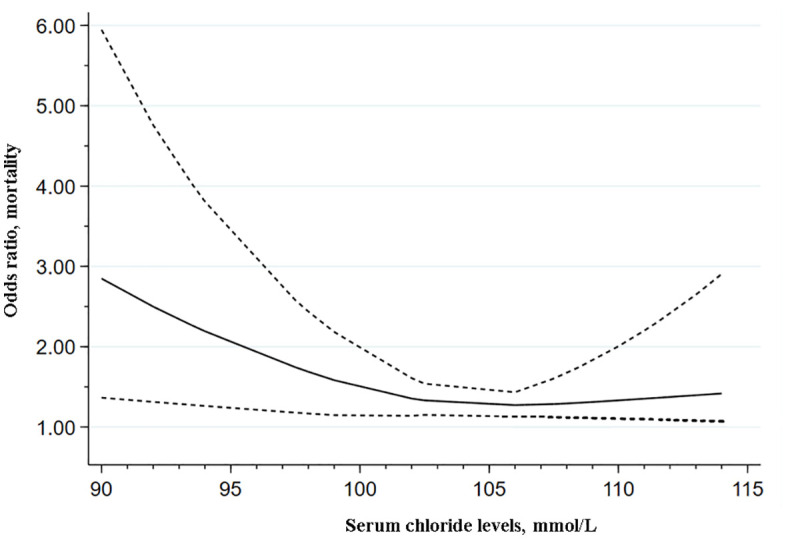
The dose response relationship between serum chloride levels and mortality risk.

Dose-response analysis incorporating 7 studies [13,42,43,48,53,58,63] revealed a significant non-linear association between serum chloride levels and mortality risk (χ² = 5.85, *p* = 0.021). As illustrated in Fig 7, the restricted cubic spline model exhibited a non-linear relationship, indicating that both hypochloremia and hyperchloremia were independent risk factors. Mortality risk increased steeply at lower concentrations, with odds ratios (ORs) of 2.85 (95% CI [1.38–5.95]), 2.05 (95% CI: 1.24–3.39), and 1.51 (95% CI [1.16–2.01]) at 90, 95, and 100 mmol/L, respectively, when referenced to 106 mmol/L. A more gradual increase in risk was observed at higher levels, with ORs of 1.34 (95% CI [1.11–2.00]) at 110 mmol/L and 1.39 (95% CI [1.07–2.90]) at 114 mmol/L. Within the normal range of chloride levels (100 mmol/L −105 mmol/L), the relationship with the risk of mortality was the lowest (S4 File).

## Discussion

Through a systematic review and meta-analysis of 34 studies (n = 175,021 critically ill patients), we comprehensively evaluated the prevalence of dysregulated serum chloride levels and its association with the risks of on mortality and AKI risk. The pooled prevalence was 34% for hyperchloremia and 14% for hypochloremia, respectively. Both conditions were significant independent risk factors for mortality, with hyperchloremia associated with a 28% increased risk and hypochloremia with a 55% increased risk of Mortality. Hyperchloremia was also associated with an elevated risk of AKI. A dose-response analysis revealed a non-linear relationship, where mortality risk was elevated at both the lower and upper extremes of serum chloride levels, confirming that both hypochloremia and hyperchloremia are independent risk factors for mortality.

The influence of serum chloride disturbances on patient outcomes has been the subject of extensive research, yet clinical understanding has largely focused on critically ill patients. Our meta-analysis revealed that the pooled prevalence of hyperchloremia (elevated serum chloride levels (typically chloride > 106 mmol/L)) among critically ill patients was 34%, a finding in line with previous reports [67]. Results from the current meta-analysis corroborate previous findings: mean serum chloride levels were notably lower in the non-survivors compared to survivors, and hyperchloremia were linked to an increased mortality risk among critically ill patients. This aligns with evidence from specific populations, such as those with acute heart failure [68,69], stable heart failure [70], and broader critical care groups [46,68,69], in which hyperchloremia consistently emerges as an independent prognostic indicator of poor outcomes. Although the exact mechanisms by which hyperchloremia leads to adverse outcomes are not fully understood, several hypotheses have been put forward. First, elevated chloride levels may over-activate the renin–angiotensin–aldosterone system (RAAS), leading to reduced renal cortical perfusion and contributing to intracapsular hypertension, potentially leading to iatrogenic hyperchloremic metabolic acidosis, which is associated with increased mortality in critically ill patients [61]. Second, hyperchloremia has paradoxical effects. It may possess anti-inflammatory properties [71] but becomes pro-inflammatory when combined with hypernatremia, a common condition in metabolic syndrome due to high dietary sodium chloride intake [72], while also increasing intra-abdominal pressure [73] and inducing coagulation disorders [74], all of which may elevate mortality risk. Third, experimental evidence demonstrates that hyperchloremia significantly impairs hemodynamics and cardiac function in animal models [8,75], a finding that aligns with clinical associations between chloride abnormalities and the increased use of vasoactive medications in adults [23,24,54]. Overall, these mechanisms highlight the complex interplay between chloride dysregulation and critical illness, although the physiological pathways underlying its clinical impact require further exploration to clarify causality and inform therapeutic strategies.

Our meta-analysis found a hypochloremia prevalence of 14%, which was consistent with the established range of 10.2%–20.9% reported in prior studies [76,77]. Furthermore, hypochloremia was identified as a significant risk factor, conferring a 55% increase in mortality. This confirms its role as an independent prognostic marker, a finding previously documented in specific cohorts (e.g., heart failure) [68,78] and in large, heterogeneous populations [57,61,77]. The pathophysiological mechanisms underlying this association have not been fully elucidated but are believed to be multifactorial. Proposed mechanisms include a direct pro-inflammatory effect, whereby a low chloride concentration may enhance cytokine release [79,80], and an indirect effect, whereby hypochloremia acts as a marker for complex severe pathologies, such as higher blood pressure [81] and heart failure [82], which increase the mortality risk [68,83]. Additionally, recent advances in characterizing chloride channels, which are central to maintaining physiological stability, suggest another plausible pathway [84].

Notably, a dose-response analysis revealed a non-linear relationship where mortality risk was elevated at both the lower and upper extremes of serum chloride levels, confirming that both hypochloremia and hyperchloremia are independent risk factors for mortality—an observation echoed by a few studies suggesting a U-shaped correlation between these chloride disturbances and adverse

outcomes [84,85]. However, our meta-analysis did not identify this U-shaped relationship, with inconsistencies in the reported associations likely stemming from heterogeneity across the included studies, such as variations in patient populations (e.g., differing disease types and ages) and the thresholds used to define hypochloremia and hyperchloremia. Despite these discrepancies, the core premise—that both hypochloremia and hyperchloremia are linked to a heightened mortality risk—has been consistently supported by previous research [40,53,58,66] and corroborated by our current meta-analytic findings. This bidirectional association underscore the pivotal role of chloride in maintaining systemic homeostasis, as deviations from normal levels in either direction disrupt interconnected physiological pathways, as outlined earlier [61,71,72,79,80]. Collectively, these findings emphasize that chloride homeostasis is not merely a secondary indicator but a key determinant of clinical outcomes, highlighting the need for targeted monitoring and management of both hypochloremia and hyperchloremia to mitigate the mortality risk in critically ill patients.

In recent years, the use of normal saline for fluid resuscitation in critically ill patients has drawn increasing concern, as it can induce hyperchloremia, which in turn elevates the risk of not only mortality but also AKI [1,9]. Our current meta-analysis, by synthesizing previous research, reveals that hyperchloremia might be associated with an increased AKI risk. Numerous studies across various ICU populations, including those with sepsis [6], diabetic ketoacidosis [86], and pancreatitis [87], have established a link between hyperchloremia and AKI. However, randomized trials comparing balanced crystalloids to normal saline for ICU fluid resuscitation have yielded inconsistent results, with no significant differences in AKI incidence reported [88,89]. A previous systematic review and meta-analysis comparing fluid resuscitation with balanced solutions versus isotonic saline in adult patients in operating rooms and ICUs also found no difference in AKI occurrence [90]. These discrepancies may arise from the complex interplay of factors influencing AKI pathogenesis. Mechanistically, chloride-induced changes in renal blood flow [91], along with evidence from animal and human studies indicating that saline infusions reduce the glomerular filtration rate, delay micturition, and impair renal perfusion [9,92], suggest a plausible role for hyperchloremia in AKI development. Nevertheless, the etiology of AKI likely involves a wide range of clinical, pathological, and epidemiological interactions [93–95], underscoring the urgent need for further research to clarify the relative impact of chloride levels and fluid choice on renal outcomes in critical care settings.

Our study had several limitations. First, there is notable heterogeneity among the included studies. Despite attempts to explore this via subgroup analyses, significant variation persisted, likely the diverse critically ill populations, patient age, and differing definitions of hypochloremia across studies. Consequently, the pooled estimates should be interpreted with caution. Second, the assessment for small-study effects (e.g., via funnel plots and Egger’s test) was limited by the small number of studies in some analyses, and its results should be interpreted with caution. Third, the prevalence estimates for hypochloremia lacks precision and generalizability due to derivation from only three studies, raising concerns about sampling bias. Furthermore, this small sample limits the generalizability of the finding to the broader critically ill population. Therefore, this result should be considered preliminary and necessitates validation in future research. Fourth, the inability to quantitatively assess the association between hypochloremia and AKI due to an insufficient number of primary studies reporting this specific outcome is a notable limitation. This represents a critical evidence gap, leaving a critical evidence gap regarding the relationship between low chloride levels and renal outcomes. Despite these limitations, our findings highlight an important clinical issue: fluctuations in chloride levels and their impact on patient outcomes are often overlooked. Emerging evidence indicates that hyperchloremia in AKI survivors may be a sign of incomplete renal recovery and an increased risk of disease progression [96]. Therefore, it is imperative that clinicians pay closer attention to monitoring serum chloride levels and understand their influence on the incidence and prognosis of AKI.

## Conclusion

In summary, this study provides the first comprehensive meta-analysis quantifying the prevalence of hypochloremia and hyperchloremia, and their links to mortality and AKI in critically ill patients. Critically, the current meta-analysis revealed that hyperchloremia is highly prevalent in critically ill patients and is a potent predictor of mortality. A non-linear relationship between serum chloride levels and mortality risk showed that both hypochloremia and hyperchloremia served as independent risk factors, underscoring the critical importance of maintaining chloride homeostasis. Hyperchloremia is also associated with an increased risk of AKI. These results underscore that chloride homeostasis is a crucial therapeutic target in critical care, and future studies should investigate the efficacy of correcting chloride abnormalities.

## Supporting information

S1 File PRISMA checklist. (PDF)

S2 File Literature search strategy. (PDF)

S3 File Threshold definitions for hyperchloremia and hypochloremia among the included studies. (PDF)

S4 File The predicted values of dose–response analysis of serum chloride levels and mortality risk in critically ill patients. (PDF)

S1 Fig Sensitivity analysis of overall prevalence of hyperchloremia. (TIF)

S2 Fig The funnel plot assessed the publication bias for overall prevalence of hyperchloremia. (TIF)

S3 Fig Sensitivity analysis of the chloride levels between survived and non-survived critically ill patients. (TIF)

S4 Fig The funnel plot assessed the publication bias for the chloride levels between survived and non-survived critically ill patients. (TIF)

S5 Fig Sensitivity analysis of the association between hyperchloremia and the risk of mortality. (TIF)

S6 Fig The funnel plot assessed the publication bias for the association between hyperchloremia and the risk of mortality. (TIF)

S7 Fig Sensitivity analysis of the association between hypochloremia and the risk of mortality. (TIF)

S8 Fig The funnel plot assessed the publication bias for the association between hypochloremia and the risk of mortality. (TIF)

S9 Fig The trim-and-fill method with funnel plot assessed the publication bias for the association between hypochloremia and the risk of mortality. (TIF)

S10 Fig Sensitivity analysis of the association between hyperchloremia and the risk of AKI. (TIF)
