# Supplementary material for: Dysregulated serum chloride and clinical outcomes in critically ill adults: A systematic review and meta-analysis
Source: PLoS One. 2025 Dec 1;20(12):e0337560. doi: 10.1371/journal.pone.0337560 (PMC12668489; doi:10.1371/journal.pone.0337560)
Supplement: S2 File — (PDF) [file pone.0337560.s002.pdf]

S2 File. Literature search strategy

Databases	Search number	Query	Records
PubMed			
	#1	"Intensive Care Units"[Mesh]	113,368
	#2	(((((((((((((((Intensive Care Unit[Title/Abstract]) OR (close attention unit[Title/Abstract])) OR (ICU[Title/Abstract])) OR (ICUs[Title/Abstract])) OR (critical care unit[Title/Abstract])) OR (GICU[Title/Abstract])) OR (GICUs[Title/Abstract])) OR (ICU`s[Title/Abstract])) OR (intensive care department[Title/Abstract])) OR (intensive therapy unit[Title/Abstract])) OR (intensive treatment unit[Title/Abstract])) OR (respiratory care unit[Title/Abstract])) OR (respiratory care units[Title/Abstract])) OR (special care unit[Title/Abstract])) OR (Critical illnesses[Title/Abstract])) OR (Critical Illnesses[Title/Abstract])) OR (Critically ill[Title/Abstract])	235,719
	#3	#1 OR #2	273,655
	#4	"Chlorides"[Mesh]	142,587
	#5	(((((((Chlorides[Title/Abstract]) OR (Chloride[Title/Abstract])) OR (Chlorhydrate[Title/Abstract])) OR (Hydrochloride[Title/Abstract])) OR (Monochloride[Title/Abstract])) OR (Chloride Ion Level[Title/Abstract])) OR (Hyperchloremia[Title/Abstract])) OR (hypochloremia[Title/Abstract])	392,971
	#6	#4 OR #5	392,971
	#7	#3 AND #7	1,624
Embase			
	#1	'intensive care unit'/exp	338,055
	#2	'intensive care units':ab,ti OR 'intensive care unit':ab,ti OR 'close attention unit':ab,ti OR icu:ab,ti OR icus:ab,ti OR 'critical care unit':ab,ti OR gicu:ab,ti OR gicus:ab,ti OR icu`s:ab,ti OR 'intensive care department':ab,ti OR 'intensive therapy unit':ab,ti OR 'intensive treatment unit':ab,ti OR 'respiratory care unit':ab,ti OR 'respiratory care units':ab,ti OR 'special care unit':ab,ti OR 'critical illnesses':ab,ti OR 'critically ill':ab,ti	401,033
	#3	#1 OR #2	519, 409
	#4	'chloride'/exp	62,495
	#5	chloride:ab,ti OR chlorhydrate:ab,ti OR hydrochloride:ab,ti OR monochloride:ab,ti OR 'chloride ion level':ab,ti OR hyperchloremia:ab,ti OR hyperchloremia:ab,ti	276,308
	#6	#4 OR #5	308,597
	#7	#3 AND #7	3,024
Cochrane			
	#1	MeSH descriptor: [Intensive Care Units] explode all trees	6,427
	#2	'intensive care units':ab,ti OR 'intensive care unit':ab,ti OR 'close attention unit':ab,ti OR icu:ab,ti OR icus:ab,ti OR 'critical care unit':ab,ti OR gicu:ab,ti OR gicus:ab,ti OR icu`s:ab,ti OR 'intensive care department':ab,ti OR 'intensive therapy unit':ab,ti OR 'intensive treatment unit':ab,ti OR 'respiratory care unit':ab,ti OR 'respiratory care units':ab,ti OR 'special care unit':ab,ti OR 'critical illnesses':ab,ti OR 'critically ill':ab,ti	46,973
	#3	#1 OR #2	47,956
	#4	MeSH descriptor: [Chlorides] explode all trees	3,495

#5	chloride:ab,ti OR chlorhydrate:ab,ti OR hydrochloride:ab,ti OR monochloride:ab,ti OR 'chloride ion level':ab,ti OR hyperchloremia:ab,ti OR hyperchloremia:ab,ti	22,023
#6	#4 OR #5	24,615
#7	#3 AND #7	2,729
Web of Science		
#1	Intensive Care Units (Topic) OR Intensive Care Unit (Topic) OR close attention unit (Topic) OR ICU (Topic) OR ICUs (Topic) OR critical care unit (Topic) OR GICU (Topic) OR GICUs (Topic) OR ICU`s (Topic) OR intensive care department (Topic) OR intensive therapy unit (Topic) OR intensive treatment unit (Topic) OR respiratory care unit (Topic) OR respiratory care units (Topic) OR special care unit (Topic) OR Critical illnesses (Topic) OR Critical Illnesses (Topic) OR Critically ill (Topic)	511,175
#2	Chlorides (Topic) OR Chloride (Topic) OR Chlorhydrate (Topic) OR Hydrochloride (Topic) OR Monochloride (Topic) OR Chloride Ion Level (Topic) OR Hyperchloremia (Topic) OR hypochloremia (Topic)	1,687,555
#3	#1 AND #2	3,320
