# Supplementary material for: Dysregulated serum chloride and clinical outcomes in critically ill adults: A systematic review and meta-analysis
Source: PLoS One. 2025 Dec 1;20(12):e0337560. doi: 10.1371/journal.pone.0337560 (PMC12668489; doi:10.1371/journal.pone.0337560)
Supplement: S3 File — (PDF) [file pone.0337560.s003.pdf]

S3 File. Threshold definitions for hyperchloremia and hypochloremia among the included studies.

Studies	Threshold definitions for Hyperchloremia and hypochloremia
Amara et al (2021)	Hyperchloremia, Chloride > 106 mmol/L
Barlow et al (2022)	Hyperchloremia, Chloride > 109 mmol/L
Huang et al (2018)	Hyperchloremia, Chloride > 110 mmol/L
Lee et al (2016)	Hyperchloremia, Chloride > 110 mmol/L
Oh et al (2018)	Hyperchloremia, Chloride > 110 mmol/L
Shao et al (2016)	Hyperchloremia, Chloride > 108 mmol/L
Regenmortel et al (2016)	Hyperchloremia, Chloride > 106 mmol/L
Yeh et al (2019)	Hyperchloremia, Chloride > 110 mmol/L
Yessayan et al (2017)	Hyperchloremia, Chloride > 110 mmol/L
Zhang et al (2024)	Hyperchloremia, Chloride > 109 mmol/L
Al Qahtani et al (2023)	Hyperchloremia, Chloride > 106 mmol/L
Tan et al (2024)	Hyperchloremia, Chloride > 108 mmol/L
Semmler et al (2022)	Hyperchloremia, Chloride > 107 mmol/L
Zhou et al (2023)	Hyperchloremia, Chloride > 110 mmol/L
Neyra et al (2015)	Hyperchloremia, Chloride > 110 mmol/L
Tani et al (2012)	Hyperchloremia, Chloride > 106 mmol/L,
Chen et al (2025)	Hyperchloremia, Chloride > 106 mmol/L; hypochloremia, Chloride < 100 mmol/L
Lei et al (2025)	Hyperchloremia, Chloride > 106 mmol/L; hypochloremia, Chloride < 99 mmol/L,
