# Supplementary material for: Dysregulated serum chloride and clinical outcomes in critically ill adults: A systematic review and meta-analysis
Source: PLoS One. 2025 Dec 1;20(12):e0337560. doi: 10.1371/journal.pone.0337560 (PMC12668489; doi:10.1371/journal.pone.0337560)
Supplement: S4 File — (PDF) [file pone.0337560.s004.pdf]

S4 File. The predicted values of dose–response analysis of serum chloride levels and mortality risk in critically ill patients.

Serum Chloride (mmol/L)	Predicted Odds Ratio (95% CI)
90	2.85 (1.38, 5.95)
95	2.05 (1.24, 3.39)
100	1.51 (1.16, 2.01)
105	1.28 (1.13, 1.46)
110	1.34 (1.11, 2.00)
114	1.39 (1.07, 2.90)
